# Clinical outcomes in idursulfase-treated patients with mucopolysaccharidosis type II: 3-year data from the hunter outcome survey (HOS)

**DOI:** 10.1186/s13023-017-0712-3

**Published:** 2017-10-03

**Authors:** Joseph Muenzer, Roberto Giugliani, Maurizio Scarpa, Anna Tylki-Szymańska, Virginie Jego, Michael Beck

**Affiliations:** 1 Department of Pediatrics, University of North Carolina at Chapel Hill, Chapel Hill, NC USA; 2 Medical Genetics Service/HCPA, Department of Genetics/UFRGS and INAGEMP, Porto Alegre, Brazil; 3 Rare Disease Centre, Helios Dr. Horst Schmidt Clinic, Wiesbaden, Germany; 4 Department of Women’s and Children’s Health, University of Padova, Padova, Italy; 5 Department of Pediatric Nutrition and Metabolic Diseases, The Children’s Memorial Health Institute, Warsaw, Poland; 6 Cytel, Inc., Geneva, Switzerland; 7 Department of Pediatrics, University Medical Center, Johannes Gutenberg University Mainz, Mainz, Germany

**Keywords:** Hunter syndrome, Lysosomal storage disease, Idursulfase, Efficacy, Disease registry

## Abstract

**Background:**

Mucopolysaccharidosis type II (MPS II; Hunter syndrome) is a rare, X-linked disorder caused by deficient activity of the enzyme iduronate-2-sulfatase (I2S). Treatment is available in the form of enzyme replacement therapy (ERT) with recombinant I2S. Clinical outcomes following ≥3 years of ERT with idursulfase were investigated in a broad population of patients with MPS II enrolled in the Hunter Outcome Survey (HOS).

**Methods:**

As of January 2016, 639 patients (excluding female patients, individuals who had received a bone marrow transplant and those enrolled in the phase 1/2 [TKT018] or phase 2/3 [TKT024] clinical trial) followed prospectively in the registry had received idursulfase for ≥6 months. These individuals all had data available for ≥1 clinical parameter at baseline and ≥1 additional time point following treatment initiation. Changes in clinical parameters were assessed in the subcohorts of patients with a measurement at baseline and at year 1, 2 or 3 of treatment. Safety data from patients who started treatment at or after enrollment in HOS (*n* = 233) were also assessed.

**Results:**

Median (10th, 90th percentiles) age at first treatment was 6.2 (2.1, 18.2) years and median treatment duration was 56.3 (18.2, 97.6) months. Urinary glycosaminoglycan (uGAG) levels decreased from baseline to year 3 in patients with data available at this time point (median change from baseline: −201.0 [−591.4, −21.9] μg/mg creatinine [*n* = 121]). Improvements in the following parameters were observed at year 3 in the subcohorts: 6-min walking test (6MWT) distance, 10.6 (−33.6, 50.8)% (*n* = 26); left ventricular mass index (LVMI), −9.3 (−31.5, 19.7)% (*n* = 52); absolute forced vital capacity (FVC), 29.7 (−13.4, 66.7)% (*n* = 23); absolute forced expiratory volume in 1 s (FEV_1_), 22.8 (−15.2, 62.1) % (*n* = 22); palpable liver size, −54.5 (−85.7, 50.0)% (*n* = 53); palpable spleen size, −33.3 (−80.0, 33.3)% (*n* = 17). No new or unexpected safety concerns were identified in this analysis.

**Conclusions:**

These findings suggest that idursulfase has a positive effect on uGAG levels, 6MWT results, LVMI, FVC, FEV_1_ and hepatosplenomegaly after 1, 2 and 3 years treatment.

**Electronic supplementary material:**

The online version of this article (10.1186/s13023-017-0712-3) contains supplementary material, which is available to authorized users.

## Background

Mucopolysaccharidosis type II (MPS II; Hunter syndrome; OMIM 309900) is a rare, X-linked recessive, life-limiting metabolic disease [1], with an estimated incidence of 0.6–1.3 in 100,000 live male births [2, 3]. It is caused by deficient activity of iduronate-2-sulfatase (I2S), a lysosomal enzyme that catalyses a step in the catabolism of glycosaminoglycan (GAG). Accumulation of GAG in nearly all tissues and organs contributes to the chronic, progressive, multisystemic clinical signs and symptoms of MPS II [1].

Although somatic signs and symptoms are present in all patients, the severity of MPS II spans a broad range [1]. Around two-thirds of patients will develop central nervous system (CNS) involvement with progressive profound cognitive impairment [4]. Death occurs in the first or second decade of life in patients with cognitive impairment who are untreated, most commonly as a result of complications associated with obstructive airway disease or cardiac failure [1, 5]. Disease progression is often slower in individuals without cognitive impairment, with patients with normal cognitive function typically surviving into adulthood [1, 5].

Significant improvements have been made in the management of MPS II over the last 2 decades, including advances in symptomatic care and the availability of disease-specific treatments such as enzyme replacement therapy (ERT) with recombinant I2S [4, 6]. Most clinical experience has been gathered with intravenously administered idursulfase (Elaprase®, Shire, Lexington, MA, USA), although other therapies for the treatment of MPS II are currently in development. Improvements in clinical parameters such as forced vital capacity (FVC) and distance walked in the 6-min walk test (6MWT), as well as decreases in liver and spleen size (as measured by magnetic resonance imaging) and urinary GAG (uGAG) levels have been demonstrated in clinical trials of intravenous idursulfase in patients with MPS II [7–9]. A recent analysis of data from the Hunter Outcome Survey (HOS) also demonstrated improved survival in patients who received idursulfase compared with those who were untreated [10]. In addition, idursulfase has been shown to be generally well tolerated, with a safety profile similar to that reported for ERT in patients with other mucopolysaccharidoses [7–9, 11–14]. However, long-term follow-up is particularly important for gaining a better understanding of disease progression and for evaluating the efficacy of ERT, particularly in routine clinical practice.

The Hunter Outcome Survey (HOS) is a large, multicentre, observational registry established in 2005 and collects long-term data on the natural history of MPS II and the efficacy and safety of ERT with idursulfase [4, 5, 15–23]. Data were analysed to investigate clinical outcomes following up to 3 years of idursulfase treatment in a broad population of patients with MPS II.

## Methods

### Registry design and data collection

HOS is designed to collect data on patients with MPS II based on information obtained during routine patient visits and assessments [4]. The registry is open to all patients with MPS II who are untreated or who are receiving treatment with idursulfase; patients receiving any other form of ERT are excluded. A wide range of information, including demographic and clinical data, is captured; however, there are no predetermined assessments in the registry. As such, patient visits and assessments occur as deemed appropriate by the treating physician and all investigations are performed using the methods and techniques commonly employed at each clinic. Data can be entered from patients who are alive at study enrollment (prospective patients), and also from individuals who are deceased at enrollment (historical patients) if local regulations permit. Before enrollment, Independent Review Board/Ethics Committee approval was obtained for all participating centres. Written informed consent was obtained from each patient, their parents or legal representative. All patient information is managed in accordance with national data protection standards.

The presence of cognitive involvement was determined based on the answer to the following question: ‘Cognitive impairment? Yes/No′ for the period from birth to HOS entry and at subsequent visits (i.e. at any time). The answer to this question is based on either the results of standardized cognitive tests, or on the clinical impression of the treating physician.

Functional classification was collected in the database based on the clinical impression of the attending physician and classed as ‘normal’ (approximate intelligence quotient [IQ] >80), ‘borderline’ (IQ 70–80), ‘educable’ (IQ 50–70), ‘trainable’ (IQ 30–50) or ‘profoundly impaired’ (IQ <30). These categories correspond to, but are not identical to, the severity levels for intellectual disability described in the Diagnostic and Statistical Manual of Mental Disorders, fourth edition (DSM-IV) (American Psychiatric Association, 2000) [24]. A functional classification of ‘educable’ in the registry corresponds to ‘mild’ in the DSM-IV (IQ 50/55–70), ‘trainable’ to ‘moderate’ (IQ 35/50–50/55), and ‘profoundly impaired’ to ‘severe’ and ‘profound’ severity (IQ <35/40).

Adverse events (AEs) are recorded in the HOS database. The seriousness of an AE (serious or non-serious), its relationship to treatment with idursulfase (not related, possibly related or probably related to treatment), as well as severity of the AE (mild, moderate or severe) can be entered in the database. An infusion-related reaction is defined in the HOS protocol as an AE that occurs during an infusion or up to 24 h post-infusion and for which there is evidence of a causal relationship with idursulfase. AEs entered in HOS are coded using the Medical Dictionary for Regulatory Activities (MedDRA; version 16.0). Events that were not MedDRA coded owing to lack of information are listed as ‘Not coded’. Coded AEs were grouped by system organ class and preferred term; patients were counted only once within each system organ class and preferred term.

### Patient population

As of January 2016, 1096 individuals (prospective and historical patients) were enrolled from 124 centres in 29 countries. Of these patients, 947 were alive at HOS entry and were followed prospectively in the registry; the remaining 149 were deceased at enrollment and had data entered retrospectively.

Male patients who had received idursulfase for at least 6 months and who were followed prospectively were included in this analysis. These individuals all had data available for at least one clinical parameter at baseline and at least one additional time point following treatment initiation. Female patients, individuals who were enrolled in the phase 1/2 (TKT018) or phase 2/3 (TKT024) trial, and those who had received a bone marrow transplant were excluded. Two individuals received treatment with intrathecally delivered idursulfase via participation in a clinical trial (SHP609-302, ClinicalTrials.gov identifier: NCT02412787) and so were no longer eligible to participate in HOS; for these patients, data after the date of surgical implantation of the intrathecal drug delivery device were not included in this analysis.

### Data analysis

Improvements in clinical parameters following ERT with idursulfase have been found to be particularly pronounced within the first year of treatment [25, 26]. Therefore, clinical and biochemical measures recorded at annual time points over 3 years (data collected at any point between study start [2005] and January 2016) were compared with baseline values. The baseline measurement was defined as the last recorded measurement in the 12 months preceding the start of idursulfase treatment.

A post-baseline measurement was defined as the value recorded closest to the 1st, 2nd or 3rd-year anniversary of the baseline measurement, within 6 months either side of the anniversary date. If there were two values within equal times of the evaluation visit, the first value was used. Selected parameters were assessed in the subcohort of patients with a measurement at baseline and at year 1, 2 or 3 of treatment. For each clinical parameter, the subcohort at year 1 comprised patients with data available on that parameter at both baseline and year 1; the subcohort at year 2 comprised patients with data available at both baseline and year 2; the subcohort at year 3 comprised patients with data available at both baseline and year 3. An additional analysis of uGAG values was performed using data only from patients who had a measurement at all time points (baseline, year 1, year 2 and year 3). uGAG values recorded in the database were obtained either from a local laboratory or from a central laboratory (analyses of uGAG levels were conducted by the Shire central laboratory between 3 October 2005 and 28 February 2014; analyses conducted after 1 March 2014 were performed by LabCorp). uGAG levels were determined by methods used in the local or central laboratories. uGAG values included in this analysis may have been determined by different methods and therefore as well as absolute changes, percentage changes from baseline are presented to give an indication of general trends. Left ventricular mass index (LVMI) was calculated from an echocardiogram using the Devereux formula [27] and adjusted for body surface area using the closest height or weight measurement taken within 91.5 days either side of the investigation date. Left ventricular hypertrophy (LVH) was defined as an LVMI greater than 102 g/m^2^. For analysis of pulmonary measures and 6MWT distance, only data from patients aged at least 5 years at the date of assessment were included. In addition, data from those patients who required assistance to complete the 6MWT were excluded. Liver and spleen sizes were obtained using palpation according to standard clinical practice at each centre [18, 28].

To investigate the incidence of serious AEs (SAEs) occurring during idursulfase treatment, data on SAEs were analysed in those patients who started ERT with idursulfase at or after enrollment in HOS (*n* = 233). AEs occurring during the period of enrollment, including a description of the event, its timing, severity and any possible or probable relationship with idursulfase (judged by the attending physician), were recorded in the registry by the clinic. Events were coded as either infusion-related or not infusion-related; the former was defined as events occurring during or within 24 h of an infusion and with evidence of a causal relationship with idursulfase.

### Statistical methods

Summary tabulations of the number and percentage of subjects within each category are presented for categorical variables. For continuous variables, the number of individuals and median values (10th and 90th percentiles) are presented. A t-test was used to test for the significance in change from baseline to year 3 between positive and negative groups in the antibody status and neutralizing antibody status groups.

## Results

### Patient population

In total, 639 male patients in HOS had received idursulfase for at least 6 months, were alive at HOS entry, had not received a bone marrow transplant and had not participated in the TKT018 or TKT024 trial. The median (10th, 90th percentiles) age at treatment start in these patients was 6.2 (2.1, 18.2) years and the median duration of treatment was 56.3 (18.2, 97.6) months. The demographic and baseline characteristics for these patients are shown in Table 1.

**Table 1:** Demographics and baseline characteristics of patients included in the analysis

	Patients, *N* = 639
Age at onset of symptoms, years (*n* = 517)	1.5 (0.3, 4.0)
Age at diagnosis, years (*n* = 582)	3.1 (1.0, 6.7)
Age at first treatment, years	6.2 (2.1, 18.2)
Age at first treatment, *n* (%)	
0–5 years	310 (48.5)
6–11 years	189 (29.6)
12–17 years	75 (11.7)
18–29 years	45 (7.0)
> 29 years	20 (3.1)
Time on treatment, months	56.3 (18.2, 97.6)
Time in HOS, months	46.8 (9.6, 93.0)
Treatment started at or after HOS entry, *n* (%)	233 (36.5)
Deceased, *n* (%)	97 (15.2)
Age at death, years (*n* = 97)	15.9 (10.6, 28.0)
Cognitive impairment (at any time), *n* (%) (*n* = 626)	
Yes	385 (61.5)
No	241 (38.5)
Last-reported functional classification, *n* (%) (*n* = 467)	
Normal	179 (38.3)
Borderline	51 (10.9)
Educable	40 (8.6)
Trainable	71 (15.2)
Profoundly impaired	126 (27.0)

Data are presented as median (10th, 90th percentiles) unless stated otherwise. Data were available for all patients included in the analysis (*N* = 639) unless stated otherwise. HOS, Hunter Outcome Survey

Cognitive impairment at any time was recorded for 61.5% (385/626) of the patients with available information. Last-reported functional classification was available for 73.1% (467/639) of patients. Of these 467 patients, 38.3% were considered ‘normal’, 10.9% ‘borderline’, 8.6% ‘educable’, 15.2% ‘trainable’ and 27.0% ‘profoundly impaired’.

A total of 97 patients died after enrollment (median [10th, 90th percentiles] age at death, 15.9 [10.6, 28.0] years). Of these individuals, 94 had information on cognitive impairment available; 75 were reported to have cognitive involvement. The most commonly reported causes of death were respiratory failure (39%; *n* = 38), causes classified as ‘other’ (35%; *n* = 34) and cardiac arrest (12%; *n* = 12). The median age at treatment start in these patients was 10.7 (6.0, 25.2) years and the median duration of treatment was 27.4 (7.5, 86.5) months.

### Clinical outcomes

**Fig. 1:**
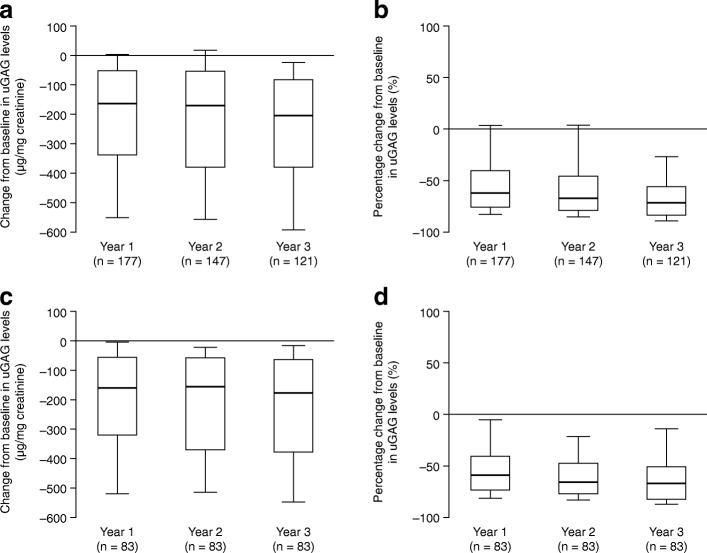
Change in urinary glycosaminoglycan (uGAG) levels. **a** Absolute change and (**b**) percentage change from baseline^a^ in uGAG levels in patients with data available after 1, 2 or 3 years of treatment with idursulfase. **c** Absolute change and (**d**) percentage change from baseline in uGAG levels in patients with data available at consecutive time points (year 1, year 2 and year 3 of treatment with idursulfase). Box plots illustrate the 25th, 50th (median) and 75th percentiles (boxes) and the 10th and 90th percentiles (whiskers). ^a^The baseline measurement was defined as the last recorded measurement in the 12 months preceding the start of idursulfase treatment. A post-baseline measurement was defined as the value recorded closest to the 1st, 2nd or 3rd-year anniversary of the baseline measurement, within 6 months either side of the anniversary date. If there were two values within equal times of the evaluation visit, the first value was used. uGAG values for each patient may have been determined by different methods; therefore, absolute changes and percentage changes from baseline in uGAG levels are presented to give an indication of general trends

**Fig. 2:**
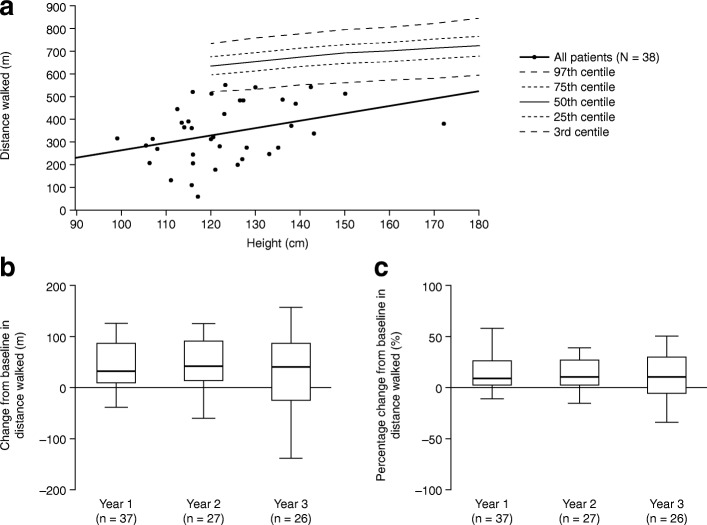
Distance walked in the 6-min walk test. **a** Baseline distance walked plotted against reference data [29] by age at assessment (*n* = 38). **b** Absolute change from baseline^a^ in distance walked in patients with data available after 1, 2 or 3 years of treatment with idursulfase. **c** Percentage change from baseline in distance walked in patients with data available after 1, 2 or 3 years of treatment with idursulfase. Box plots illustrate the 25th, 50th (median) and 75th percentiles (boxes) and the 10th and 90th percentiles (whiskers). ^a^The baseline measurement was defined as the last recorded measurement in the 12 months preceding the start of idursulfase treatment. A post-baseline measurement was defined as the value recorded closest to the 1st, 2nd or 3rd-year anniversary of the baseline measurement, within 6 months either side of the anniversary date. If there were two values within equal times of the evaluation visit, the first value was used

**Fig. 3:**
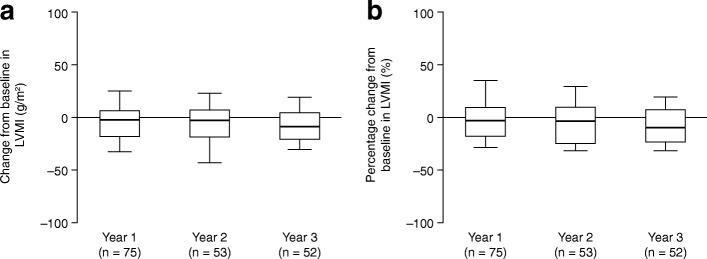
Change in left ventricular mass index (LVMI). **a** Absolute change from baseline^a^ in LVMI in patients with data available after 1, 2 or 3 years of treatment with idursulfase. **b** Percentage change from baseline in LVMI in patients with data available after 1, 2 or 3 years of treatment with idursulfase. Box plots illustrate the 25th, 50th (median) and 75th percentiles (boxes) and the 10th and 90th percentiles (whiskers). ^a^The baseline measurement was defined as the last recorded measurement in the 12 months preceding the start of idursulfase treatment. A post-baseline measurement was defined as the value recorded closest to the 1st, 2nd or 3rd anniversary of the baseline measurement, within 6 months either side of the anniversary date. If there were two values within equal times of the evaluation visit, the first value was used

**Fig. 4:**
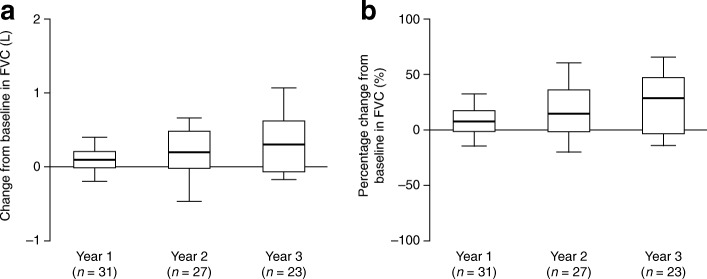
Change in forced vital capacity (FVC). **a** Absolute change from baseline^a^ in FVC in patients with data available after 1, 2 or 3 years of treatment with idursulfase. **b** Percentage change from baseline in FVC in patients with data available after 1, 2 or 3 years of treatment with idursulfase. Box plots illustrate the 25th, 50th (median) and 75th percentiles (boxes) and the 10th and 90th percentiles (whiskers). ^a^The baseline measurement was defined as the last recorded measurement in the 12 months preceding the start of idursulfase treatment. A post-baseline measurement was defined as the value recorded closest to the1st, 2nd or 3rd-year anniversary of the baseline measurement, within 6 months either side of the anniversary date. If there were two values within equal times of the evaluation visit, the first value was used

**Fig. 5:**
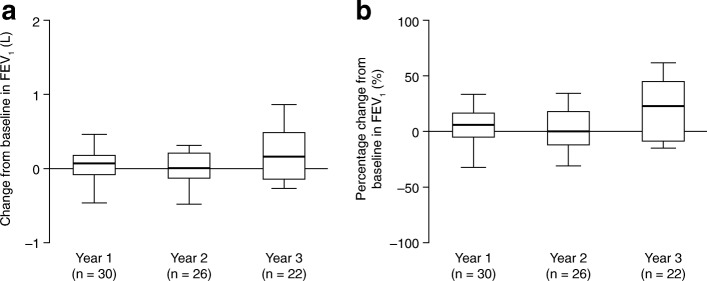
Change in forced expiratory volume in 1 s (FEV_1_). **a** Absolute change from baseline^a^ in FEV_1_ in patients with data available after 1, 2 or 3 years of treatment with idursulfase. **b** Percentage change from baseline in FEV_1_ in patients with data available after 1, 2 or 3 years of treatment with idursulfase. Box plots illustrate the 25th, 50th (median) and 75th percentiles (boxes) and the 10th and 90th percentiles (whiskers). ^a^The baseline measurement was defined as the last recorded measurement in the 12 months preceding the start of idursulfase treatment. A post-baseline measurement was defined as the value recorded closest to the 1st, 2nd or 3rd-year anniversary of the baseline measurement, within 6 months either side of the anniversary date. If there were two values within equal times of the evaluation visit, the first value was used

**Fig. 6:**
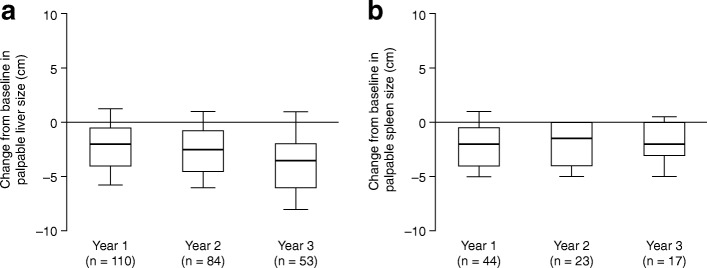
Change in palpable liver and spleen size. **a** Absolute change from baseline^a^ in palpable liver size in patients with data available after 1, 2 or 3 years of treatment with idursulfase. **b** Absolute change from baseline^a^ in palpable spleen size in patients with data available after 1, 2 or 3 years of treatment with idursulfase. Box plots illustrate the 25th, 50th (median) and 75th percentiles (boxes) and the 10th and 90th percentiles (whiskers). ^a^The baseline measurement was defined as the last recorded measurement in the 12 months preceding the start of idursulfase treatment. A post-baseline measurement was defined as the value recorded closest to the 1st, 2nd or 3rd-year anniversary of the baseline measurement, within 6 months either side of the anniversary date. If there were two values within equal times of the evaluation visit, the first value was used

Changes in clinical parameters were assessed in the subcohort of patients for whom measurements at baseline and at year 1 or 2 or 3 of treatment were available. It is important to note that the sample sizes at each time point differed and the number of patients with baseline and post-baseline data available was low for some parameters. The number of patients with data available at the three time points for the various parameters assessed ranged from 17 to 177 (as specified in Figs. 1, 2, 3, 4, 5 and 6 and Additional file 1: Table S1).

#### Change in uGAG levels

uGAG values were determined by either a local laboratory or a central laboratory using the standard method used at that laboratory. Median uGAG levels were consistently decreased from baseline at all three time points following treatment with idursulfase (Fig. 1a, Additional file 1: Table S1A). The median (10th, 90th percentiles) change in uGAG levels at year 1, year 2 and year 3 was −160.6 (−550.0, 4.2), −166.3 (−555.3, 18.5) and −201.0 (−591.4, −21.9) μg/mg creatinine, respectively. Of the individuals included in this analysis, 83 had data on uGAG levels available at all time points (years 1, 2 and 3); a similar trend in change from baseline in uGAG levels was observed in this subgroup of patients (Fig. 1c, Additional file 2: Table S2).

#### 6MWT

Baseline 6MWT distance was typically less than that of an age-matched reference population (Fig. 2a) [29]. Median distance walked was increased compared with baseline for each subcohort (i.e. year 1, year 2 and year 3) (Fig. 2b & c, Additional file 1: Table S1B). In those patients with data available at year 3 (*n* = 26), the median change from baseline was 41.0 (−138.0, 158.0) m, corresponding to a median percentage change from baseline of 10.6 (−33.6, 50.8)%.

#### LVMI

There was a reduction from baseline in LVMI at each year of treatment (Fig. 3, Additional file 1: Table S1C). In those patients with year 3 data (*n* = 52), the median (10th, 90th percentiles) change from baseline was −8.5 (−30.5, 19.3) g/m^2^, corresponding to a median percentage change from baseline of −9.3 (−31.5, 19.7)%. In patients without LVH at baseline, LVMI did not increase compared with baseline over the 3 years of treatment (year 3, −2.8 [−25.3, 58.7] g/m^2^; *n* = 40; median age, 8.4 [5.2, 17.3] years). Of the 40 patients without LVH at baseline and for whom data following 3 years of treatment were available, six were reported to have LVH at year 3. For patients with LVH at baseline, there was a general trend towards decreased LVMI with treatment, although patient numbers were limited (year 3: −23.5 [−67.7, −9.9] g/m^2^, *n* = 12; median age, 13.4 [9.0, 24.2] years).

#### Absolute FVC and FEV_1_

There was an increase from baseline in absolute FVC at each year of treatment (Fig. 4, Additional file 1: Table S1D). The median (10th, 90th percentiles) change from baseline in the year 3 subcohort was 0.3 (−0.2, 1.1) L (*n* = 23), corresponding to a median change from baseline of 29.7 (−13.4, 66.7)%. Absolute FEV_1_ was also increased at years 1 and 3, compared with baseline (Fig. 5, Additional file 1: Table S1E). In the patients with year 3 data, the median increase was 0.2 (−0.3, 0.9) L (*n* = 22), corresponding to a median change from baseline of 22.8 (−15.2, 62.1)%.

#### Palpable liver and spleen sizes

Liver and spleen sizes were measured by palpation according to standard clinical practice at each centre. Of the 165 patients for whom data on palpable liver size was available at baseline and year 3, and whose liver was palpable at baseline, 103 (62.4%) no longer had a palpable liver after treatment at year 3. Reductions in liver size compared with baseline were observed at each time point for those patients whose liver was palpable (Fig. 6a, Additional file 1: Table S1F). In those with data at year 3 (*n* = 53), the median (10th, 90th percentiles) palpable liver size at baseline was 6.0 (3.0, 11.0) cm and the median change from baseline was −3.5 (−8.0, 1.0) cm. Of the 108 patients for whom data on palpable spleen size was available at baseline and year 3, and whose spleen was palpable at baseline, 85 (78.7%) no longer had a palpable spleen after treatment at year 3. Spleen size was found to have decreased compared with baseline at each time point in those patients whose spleen was palpable (Fig. 6b, Additional file 1: Table S1G). In those with data available after 3 years of treatment (*n* = 17), the median palpable spleen size at baseline was 5.0 (1.5, 9.0) cm and the median change from baseline was −2.0 (−5.0, 0.5) cm.

### Safety analyses

Of the patients included in this analysis, 233 started treatment at or after HOS entry. A total of 1910 AEs were reported in 174 (74.7%)) of these patients between treatment start and the period of follow-up recorded in the registry. A total of 80 patients (34.3%) experienced at least one drug-related AE, 81 (34.8%) experienced at least one infusion-related AE and 34 (14.6%) experienced at least one severe AE. The most commonly reported AEs were those classified as infections and infestations, followed by respiratory, thoracic and mediastinal disorders. Of the AEs, 67.6% (1291/1910) were considered not related to treatment, 14.3% (273/1910) were considered probably related to treatment and 11.9% (227/1910) possibly related to treatment. Information on relationship to treatment was missing for 6.2% (119/1910) AEs. Of the 273 events considered probably related to treatment, 139 were mild in severity, 88 were moderate, 21 were severe and 8 were life-threatening. Of the 227 events classed as possibly related to treatment, 127 were considered mild in severity, 77 were moderate, 6 were severe and 7 were considered life-threatening. Of the 1910 AEs reported, 513 (26.9%) were recorded as infusion-related reactions; system organ class and preferred term for these are shown in Additional file 3: Table S3.

## Discussion

This study is the first analysis of real-world data from a large, broad population of patients on clinical outcomes following up to 3 years of treatment with idursulfase. Our results show improvements in uGAG levels, 6MWT distance, LVMI, absolute FVC, absolute FEV_1_ and palpable liver and spleen size compared with baseline after 1, 2 and 3 years of treatment. In addition, the results of an analysis of AEs in patients who started treatment at or after enrolment in the registry were consistent with the known safety profile of idursulfase, and two-thirds of the AEs reported were not considered related to treatment. Overall, this study suggests that idursulfase treatment has a positive effect on somatic disease symptoms in patients with MPS II in routine clinical practice.

The clinical parameters analysed were selected to match the endpoints studied in the phase 2/3 clinical trial of intravenous idursulfase in patients with MPS II, the 2-year open-label extension period, as well as a phase 4 open-label single-arm study of idursulfase treatment in boys with MPS II who were less than 5 years of age [7–9]. Our findings are broadly in line with the results from these trials and other smaller studies [25, 26, 30–34]. In particular, we found that distance walked in the 6MWT was increased compared with baseline at each time point in the analysis populations and there was also an increase from baseline in absolute FVC and FEV_1_. Although the numbers of patients with data available at year 3 for these parameters (6MWT, *n* = 26; FVC, *n* = 23; FEV_1_, *n* = 22) are lower than the number of patients included in the open-label extension period of the phase 2/3 trial (*n* = 94), our results show that idursulfase has positive effects when administered in a routine clinical setting. It is important to note that FVC and FEV_1_ are known to increase with height and age [35]. Several studies have demonstrated that ERT has a positive influence on height [8, 22] and so the improvements observed in our analysis may be due to the growth of the patients and the effect of ERT.

We observed decreases in uGAG levels compared with baseline at all time points studied, and a similar result was also seen in a subgroup of patients who had data available at consecutive time points (years 1, 2 and 3 of treatment). It should be noted that there are several limitations associated with the uGAG data presented in our analysis. First, uGAG values recorded in HOS are not all determined by the same laboratory and as such, the values reported for each patient may have been determined by different methods. In addition, uGAG levels are known to vary with age [36], and the ages of the patients included in this analysis with available data on uGAGs ranged from 0.0 to 48.0 years at baseline (*n* = 242). As a result it was not possible to analyse the changes in uGAG levels in relation to established reference ranges. Finally, it is possible that the analysis of uGAG levels may have been biased towards those patients with less severe disease, as the collection of urine (and its subsequent analysis) may be more difficult in patients with severe cognitive impairment. Nonetheless our findings do show a general trend towards decreased uGAG levels in response to treatment, which is consistent with another study of 27 paediatric patients treated with idursulfase for 3.5 years outside of a clinical trial setting [25].

Consistent with findings from the phase 1/2 study and other small-scale studies on the effectiveness of idursulfase [26, 30, 32, 34], we observed improvements in LVMI compared with baseline with each year of treatment in our analysis. Although the patient numbers in our analysis were too small to mathematically evaluate any formal relationship between the development of hypertrophic cardiomyopathy and treatment with idursulfase, it is recognized that this condition is frequently reported in untreated patients with mucopolysaccharidoses, and in MPS II in particular [37]. The literature demonstrates that cardiomyopathy is part of the underlying manifestation of MPS II and not a likely complication of ERT with idursulfase [37].

Palpable liver and spleen sizes were also found to be reduced compared with baseline at every time point studied, following idursulfase treatment. In addition, the liver and spleen were no longer palpable in significant proportions of patients at each time point. Although the estimation of liver and spleen size by palpation is standard clinical practice in the management of patients with MPS II [18, 28, 38], other methods are available that facilitate the determination of organ size with greater accuracy. These include magnetic resonance imaging and abdominal ultrasound [7–9, 25]. While the data collected in HOS can be used to provide only general information on changes in liver and spleen size, this still provides valuable information on the effects of idursulfase treatment upon this common clinical manifestation of MPS II.

While our results show that there was a general trend for improvement in all parameters assessed, we did observe variation between patients in the changes from baseline in each parameter assessed, similar to findings reported by Tomanin et al. [25]. It has been suggested that ERT should be initiated as soon as possible, to improve outcomes before significant disease progression occurs [39]. Some of the variation seen in our study may reflect differences in the age at start of treatment, since once onset of clinical disease occurs it may be difficult to stabilize or prevent progression. Additional contributing factors include the significant clinical and genetic heterogeneity typically observed in MPS II and the influence of genotype in determining the response to treatment. The investigation of genetic factors was beyond the scope of our analysis. However, it will be important to investigate the influence of this beyond the partial findings reported by Barbier et al. [40], as well as the impact of other factors on the effectiveness of ERT in the future.

This study provides valuable real-world data on ERT with idursulfase; however, there are several further limitations to consider. Unlike a formal clinical trial, there are no predetermined assessments in the registry and only information collected during routine clinical practice is included. As a result, the methods and techniques used for clinical assessments and laboratory assays are not standardized and there may also be regional variation in the frequency of follow-up visits and the investigations performed. It is also important to note that the assessment of cognitive impairment recorded in HOS varies between sites and is based on either the results of standardized cognitive tests or on the clinical impression of the treating physician. In addition, the voluntary, non-interventional nature of HOS means that not all patients included in this analysis had data available at baseline and at each time point for each of the clinical parameters investigated. For this reason, the number of patients with data available was low for some parameters, and patients whose data were included in the analyses for each time point for any given parameter were not all from the same cohort. Therefore, with the exception of the analysis of the 83 patients who had data on uGAG levels at all time points, it is not possible to directly compare results from years 1, 2 and 3 of treatment and draw any conclusions regarding sustained benefits.

It is also important to note that the number of patients with data available was low for some parameters, particularly at year 3. One reason for this is that assessments such as pulmonary function tests and 6MWT distance are not suitable for particular patient subgroups (e.g. those <5 years of age and individuals with progressive cognitive impairment) [9, 23, 41, 42]. However, almost half (48.5%) of the patients included in this analysis started treatment when they were less than 6 years old, and almost two thirds (61.5%) were reported to have cognitive impairment and so would not have had these types of tests. This highlights the need for other assessments of clinical effectiveness that can be used for these individuals.

Despite these limitations, the results from this analysis provide key insights into the impact of idursulfase treatment on clinical manifestations of MPS II during the first 3 years of treatment, and complement and extend the findings from the formal clinical trials and other smaller-scale studies of idursulfase treatment. Given the progressive nature and considerable variability in disease severity of MPS II, stabilization of certain signs and symptoms of the disease may indicate a positive effect of treatment, and data collected in registries should continue to help to increase our understanding of the benefits of ERT.

## Conclusions

This analysis of HOS data suggests that idursulfase has a positive effect on GAG storage (as evidenced by decreases in uGAG levels and hepatosplenomegaly), as well as 6MWT, LVMI, FVC and FEV_1_ results after up to 3 years of treatment. This indicates that findings from clinical trials are also seen in routine clinical practice. Long-term follow-up is particularly important for progressive disorders such as MPS II and the data collected in registries are playing an increasingly significant role in providing insights into long-term treatment effects. In the future, as the data become available, it will be important to investigate the clinical outcomes of idursulfase treatment in patients enrolled in HOS over longer periods of follow-up.

## Additional files

Additional file 1: **Table S1.** Changes from baseline in clinical and biochemical parameters in patients with data available after 1, 2 or 3 years of treatment with idursulfase (DOCX 33 kb)

Additional file 2: **Table S2.** Median urinary glycosaminoglycan (uGAG) levels and median changes from baseline in uGAG levels in patients with data available at consecutive timepoints (year 1, year 2 and year 3 of treatment with idursulfase) (DOCX 22 kb)

Additional file 3: **Table S3.** Summary of adverse events reported in patients who started treatment at or after Hunter Outcome Survey (HOS) entry (DOCX 25 kb)

## Abbreviations

6MWT: 6-min walk test
AE: Adverse event
DSM-IV: Diagnostic and Statistical Manual of Mental Disorders, fourth edition
ERT: Enzyme replacement therapy
FEV_1_: Forced expiratory volume in 1 s
FVC: Forced vital capacity
GAG: Glycosaminoglycan
HOS: Hunter Outcome Survey
I2S: Iduronate-2-sulfatase
IQ: Intelligence quotient
LVH: Left ventricular hypertrophy
LVMI: Left ventricular mass index
MedDRA: Medical Dictionary for Regulatory Activities
MPS II: Mucopolysaccharidosis type II
SAE: Serious AE
uGAG: urinary GAG
