# Supplementary material for: Clinical outcomes in idursulfase-treated patients with mucopolysaccharidosis type II: 3-year data from the hunter outcome survey (HOS)
Source: Orphanet J Rare Dis. 2017 Oct 3;12:161. doi: 10.1186/s13023-017-0712-3 (PMC5627440; doi:10.1186/s13023-017-0712-3)
Supplement: Supplementary file 1 — Table S1. Changes from baseline in clinical and biochemical parameters in patients with data available after 1, 2 or 3 years of treatment with idursulfase (DOCX 33 kb) [file 13023_2017_712_MOESM1_ESM.docx]

**Additional File 1**

**Table S1** Changes from baseline^a^ in clinical and biochemical parameters in patients with data available after 1, 2 or 3 years of treatment with idursulfase. Data are shown as median (10th, 90th percentiles).

(A) Median urinary glycosaminoglycan (uGAG) levels (µg/mg creatinine) and median changes from baseline in uGAG levels

|  | **Age (years)** | **Baseline (µg/mg creatinine)** | **Post-baseline (µg/mg creatinine)** | **Absolute change from baseline  (µg/mg creatinine)^b^** | **Change from baseline (%)** |
| --- | --- | --- | --- | --- | --- |
| **Year 1**  **(*n* = 177)** | 7.0  (2.9, 18.2) | 276.3  (72.0, 782.3) | 120.0  (27.0, 308.0) | –160.6  (–550.0, 4.2) | –61.5  (–82.6, 3.6) |
| **Year 2**  **(*n* = 147)** | 7.9  (3.6, 18.2) | 299.2  (72.0, 770.0) | 126.7  (21.2, 278.9) | –166.3  (–555.3, 18.5) | –67.1  (–84.8, 4.2) |
| **Year 3**  **(*n* = 121)** | 8.9  (4.7, 19.3) | 298.0  (72.0, 770.0) | 88.0  (21.0, 237.6) | –201.0  (–591.4, –21.9) | –71.0  (–88.2, –26.4) |

(B) Median distance (m) walked in 6-minute walk test (6MWT) and median changes from baseline in distance walked

|  | **Age  (years)** | **Baseline  (m)** | **Post-baseline (m)** | **Change from baseline (m)** | **Change from baseline (%)** |
| --- | --- | --- | --- | --- | --- |
| **Year 1**  **(*n* = 37)** | 12.6  (8.1, 22.1) | 360.0  (207.0, 518.0) | 400.0  (241.5, 550.0) | 33.0  (–39.0, 126.0) | 9.1  (–10.3, 57.9) |
| **Year 2**  **(*n* = 27)** | 13.8  (9.0, 35.9) | 379.0  (207.0, 518.0) | 422.0  (291.0, 519.0) | 42.0  (–60.0, 126.0) | 10.8  (–15.2, 39.3) |
| **Year 3**  **(*n* = 26)** | 14.6  (9.9, 39.7) | 384.5  (268.0, 518.0) | 411.0  (249.0, 545.0) | 41.0  (–138.0, 158.0) | 10.6  (–33.6, 50.8) |

(C) Median left ventricular mass index (LVMI) values (g/m^2^) and median changes from baseline in LVMI

|  | **Age (years)** | **Baseline** (**g/m^2^)** | **Post-baseline (g/m^2^)** | **Change from baseline (g/m^2^)** | **Change from baseline (%)** |
| --- | --- | --- | --- | --- | --- |
| **Year 1**  **(*n* = 75)** | 7.7  (3.8, 18.6) | 87.3  (52.4, 135.8) | 81.9  (53.3, 122.4) | –2.0  (–32.4, 25.3) | –3.2  (–28.1, 35.5) |
| **Year 2**  **(*n* = 53)** | 9.6  (4.9, 27.4) | 88.0  (54.3, 135.8) | 79.7  (55.7, 131.3) | –2.5  (–43.0, 23.2) | –3.1  (–31.0, 29.7) |
| **Year 3**  **(*n* = 52)** | 9.1  (5.5, 19.9) | 84.5  (58.3, 134.4) | 76.2  (52.1, 126.6) | –8.5  (–30.5, 19.3) | –9.3  (–31.5, 19.7) |

(D) Median forced vital capacity (FVC) values (L) and median changes from baseline in FVC

|  | **Age (years)** | **Baseline (L)** | **Post-baseline (L)** | **Change from baseline (L)** | **Change from baseline (%)** |
| --- | --- | --- | --- | --- | --- |
| **Year 1**  **(*n* = 31)** | 13.3  (7.1, 35.0) | 1.4  (0.7, 3.0) | 1.8  (0.8, 3.0) | 0.1  (–0.2, 0.4) | 8.8  (–14.0, 33.3) |
| **Year 2**  **(*n* = 27)** | 17.1  (8.0, 39.4) | 1.5  (0.7, 3.0) | 1.5  (1.0, 3.5) | 0.2  (–0.5, 0.7) | 15.3  (–19.7, 61.3) |
| **Year 3**  **(*n* = 23)** | 15.5  (10.8, 37.2) | 1.6  (0.7, 3.3) | 2.1  (0.8, 3.6) | 0.3  (–0.2, 1.1) | 29.7  (–13.4, 66.7) |

(E) Median forced expiratory volume in 1 second (FEV_1_) values (L) and median changes from baseline in FEV_1_

|  | **Age (years)** | **Baseline (L)** | **Post-baseline (L)** | **Change from baseline (L)** | **Change from baseline (%)** |
| --- | --- | --- | --- | --- | --- |
| **Year 1**  **(*n* = 30)** | 13.4  (7.1, 37.6) | 1.3  (0.6, 2.2) | 1.3  (0.5, 2.4) | 0.1  (–0.5, 0.5) | 5.8  (–32.2, 33.0) |
| **Year 2**  **(*n* = 26)** | 17.7  (8.0, 39.4) | 1.2  (0.5, 2.2) | 1.1  (0.5, 2.8) | 0.0  (–0.5, 0.3) | 0.0  (–31.2, 34.1) |
| **Year 3**  **(*n* = 22)** | 17.4  (11.4, 37.2) | 1.3  (0.7, 1.9) | 1.4  (0.7, 2.8) | 0.2  (–0.3, 0.9) | 22.8  (–15.2, 62.1) |

(F) Median palpable liver size (cm) and median changes from baseline in palpable liver size

|  | **Age (years)** | **Baseline (cm)** | **Post-baseline (cm)** | **Change from baseline** |
| --- | --- | --- | --- | --- |
| **Year 1**  **(*n* = 110)** | 7.6  (3.5, 15.3) | 5.8  (3.0, 10.6) | 3.0  (1.0, 7.5) | –2.0  (–5.8, 1.3) |
| **Year 2**  **(*n* = 84)** | 9.1  (4.6, 17.1) | 6.0  (3.0, 10.0) | 3.0  (1.0, 7.0) | –2.5  (–6.0, 1.0) |
| **Year 3**  **(*n* = 53)** | 10.8  (5.8, 21.8) | 6.0  (3.0, 11.0) | 3.0  (1.0, 6.0) | –3.5  (–8.0, 1.0) |

(G) Median palpable spleen size (cm) and median changes from baseline in palpable spleen size

|  | **Age (years)** | **Baseline (cm)** | **Post-baseline (cm)** | **Change from baseline** |
| --- | --- | --- | --- | --- |
| **Year 1**  **(n = 44)** | 9.9  (3.7, 18.0) | 5.8  (2.0, 9.3) | 3.0  (1.0, 8.0) | –2.0  (–5.0, 1.0) |
| **Year 2**  **(n = 23)** | 11.9  (5.2, 15.3) | 6.0  (2.0, 9.0) | 2.0  (1.0, 6.0) | –1.5  (–5.0, 0.0) |
| **Year 3**  **(n = 17)** | 11.8  (5.3, 15.0) | 5.0  (1.5, 9.0) | 2.0  (1.0, 7.8) | –2.0  (–5.0, 0.5) |

^a^The baseline measurement was defined as the last recorded measurement in the 12 months preceding the start of idursulfase treatment. A post-baseline measurement was defined as the value recorded closest to the 1st, 2nd or 3rd-year anniversary of the baseline measurement, within 6 months either side of the anniversary date. If there were two values within equal times of the evaluation visit, the first value was used.

^b^uGAG values for each patient may have been determined by different methods, therefore absolute changes and percentage changes from baseline in uGAG levels are presented to give an indication of general trends.
