# Supplementary material for: Clinical outcomes in idursulfase-treated patients with mucopolysaccharidosis type II: 3-year data from the hunter outcome survey (HOS)
Source: Orphanet J Rare Dis. 2017 Oct 3;12:161. doi: 10.1186/s13023-017-0712-3 (PMC5627440; doi:10.1186/s13023-017-0712-3)
Supplement: Supplementary file 2 — Table S2. Median urinary glycosaminoglycan (uGAG) levels and median changes from baseline in uGAG levels in patients with data available at consecutive timepoints (year 1, year 2 and year 3 of treatment with idursulfase) (DOCX 22 kb) [file 13023_2017_712_MOESM2_ESM.docx]

**Additional File 2**

**Table S2** Median urinary glycosaminoglycan (uGAG) levels (µg/mg creatinine) and median changes from baseline^a^ in uGAG levels in patients with data available at consecutive timepoints (year 1, year 2 and year 3 of treatment with idursulfase). Data are shown as median (10th, 90th percentiles).

|  | **Age  (years)** | **Baseline (µg/mg creatinine)** | **Post-baseline (µg/mg creatinine)** | **Absolute change from baseline (µg/mg creatinine)^b^** | **Change from baseline, %** |
| --- | --- | --- | --- | --- | --- |
| **Year 1**  **(*n* = 83)** | 6.9  (2.5, 15.9) | 291.5  (72.0, 748.8) | 124.0  (33.6, 332.6) | –160.6  (–519.2, –6.2) | –58.8  (–81.2, –5.0) |
| **Year 2**  **(*n* = 83)** | 7.9  (3.6, 16.8) | 291.5  (72.0, 748.8) | 126.7  (27.8, 271.9) | –157.0  (–513.5, –24.3) | –65.4  (–82.7, –21.3) |
| **Year 3**  **(*n* = 83)** | 8.9  (4.6, 18.0) | 291.5  (72.0, 748.8) | 105.6  (23.0, 245.5) | –177.8  (–545.6, –16.8) | –66.3  (–86.9, –13.6) |

^a^The baseline measurement was defined as the last recorded measurement in the 12 months preceding the start of idursulfase treatment. A post-baseline measurement was defined as the value recorded closest to the 1st, 2nd or 3rd-year anniversary of the baseline measurement, within 6 months either side of the anniversary date. If there were two values within equal times of the evaluation visit, the first value was used.

^b^uGAG values for each patient may have been determined by different methods, therefore absolute changes and percentage changes from baseline in uGAG levels are presented to give an indication of general trends.
