# Supplementary material for: Clinical outcomes in idursulfase-treated patients with mucopolysaccharidosis type II: 3-year data from the hunter outcome survey (HOS)
Source: Orphanet J Rare Dis. 2017 Oct 3;12:161. doi: 10.1186/s13023-017-0712-3 (PMC5627440; doi:10.1186/s13023-017-0712-3)
Supplement: Supplementary file 3 — Table S3. Summary of adverse events reported in patients who started treatment at or after Hunter Outcome Survey (HOS) entry (DOCX 25 kb) [file 13023_2017_712_MOESM3_ESM.docx]

**Additional file 3**

**Table S3.** Summary of adverse events reported in patients who started treatment at or after Hunter Outcome Survey (HOS) entry.

|  | **Adverse events** | | | |
| --- | --- | --- | --- | --- |
|  | **IRRs (*n* = 513)** | | **Non-IRRs (*n* = 1397)** | |
|  | **Events, *n* (%)** | **Patients, *n* (%)** | **Events, *n* (%)** | **Patients, *n* (%)** |
| **Total** | 513 (100.0) | 81 (34.8) | 1397 (100.0) | 159 (68.2) |
| **System organ class** |  |  |  |  |
| Blood and lymphatic system disorders | 1 (0.2) | 1 (0.4) | 9 (0.6) | 4 (1.7) |
| Cardiac disorders | 14 (2.7) | 11 (4.7) | 45 (3.2) | 26 (11.2) |
| Congenital, familial and genetic disorders |  |  | 17 (1.2) | 13 (5.6) |
| Ear and labyrinth disorders | 3 (0.6) | 3 (1.3) | 23 (1.6) | 15 (6.4) |
| Eye disorders | 5 (1.0) | 5 (2.1) | 9 (0.6) | 6 (2.6) |
| Gastrointestinal disorders | 24 (4.7) | 13 (5.6) | 115 (8.2) | 52 (22.3) |
| General disorders and administration site conditions | 48 (9.4) | 25 (10.7) | 139 (9.9) | 67 (28.8) |
| Hepatobiliary disorders |  |  | 5 (0.4) | 4 (1.7) |
| Immune system disorders | 4 (0.8) | 4 (1.7) | 2 (0.1) | 1 (0.4) |
| Infections and infestations | 13 (2.5) | 10 (4.3) | 292 (20.9) | 87 (37.3) |
| Injury, poisoning and procedural complications | 168 (32.7) | 69 (29.6) | 46 (3.3) | 27 (11.6) |
| Investigations | 17 (3.3) | 10 (4.3) | 36 (2.6) | 23 (9.9) |
| Metabolism and nutrition disorders |  |  | 30 (2.1) | 23 (9.9) |
| Musculoskeletal and connective tissue disorders | 3 (0.6) | 2 (0.9) | 55 (3.9) | 32 (13.7) |
| Neoplasms benign, malignant and unspecified |  |  | 3 (0.2) | 3 (1.3) |
| Nervous system disorders | 16 (3.1) | 14 (6.0) | 133 (9.5) | 53 (22.7) |
| Psychiatric disorders | 16 (3.1) | 7 (3.0) | 49 (3.5) | 21 (9.0) |
| Renal and urinary disorders |  |  | 9 (0.6) | 4 (1.7) |
| Reproductive system and breast disorders | 2 (0.4) | 1 (0.4) | 2 (0.1) | 2 (0.9) |
| Respiratory, thoracic and mediastinal disorders | 33 (6.4) | 17 (7.3) | 252 (18.0) | 80 (34.3) |
| Skin and subcutaneous tissue disorders | 124 (24.2) | 50 (21.5) | 35 (2.5) | 23 (9.9) |
| Social circumstances |  |  | 2 (0.1) | 2 (0.9) |
| Surgical and medical procedures | 2 (0.4) | 1 (0.4) | 76 (5.4) | 39 (16.7) |
| Vascular disorders | 20 (3.9) | 12 (5.2) | 13 (0.9) | 13 (5.6) |

The percentages of patients are based on the total number of patients who started treatment at or after HOS entry (*N* = 233); the percentages of events are based on the total number of infusion-related reactions (IRRs) or non-IRRs reported. Events were coded using the Medical Dictionary for Regulatory Activities (MedDRA; version 16.0). Patients were counted only once within each system organ class.
